# Profiling Bilateral Skills in High-Performance Male and Female Gaelic Footballers

**DOI:** 10.1177/00315125241238307

**Published:** 2024-03-23

**Authors:** Karol Dillon, Ian Sherwin, Philip E. Kearney

**Affiliations:** Sport and Human Performance Research Centre, 8808 University of Limerick, Limerick, Ireland

**Keywords:** notational analysis, bilaterality, skill assessment, dominant, non-dominant

## Abstract

Bilateral skill symmetry in sport refers to an individual’s ability to successfully perform sporting actions with both sides of the body. Two scarcely researched areas in relation to bilateral skills are the effects of opposition proximity on skill execution and bilateral skill in high-performance female athletes. In this study, we used Nacsport to code all skill executions (hand pass, kick pass, hop, solo and shot) during 121 games by both male and female participants (76 male, 45 female), classified as Tier 1 (*n* = 181, 134) and Tier 2 (*n* = 238, 115) high performance, adult Gaelic Football players. Irrespective of the participants’ tier group or gender, these players relied upon their dominant side for most skill executions (Kick Pass Dominant Foot *Mdn*: MT1 = 90%, MT2 = 98.6%, FT1 = 100%, FT2 = 100%; Solo Dominant Foot: *Mdn* MT1 = 95%, MT2 = 97.3%, FT1: 100%, FT2: 100%; Hand Pass Dominant Hand *Mdn*: MT1: 83.7%, MT2: 99%, FT1: 95.5%, FT2: 95.5%; Hop Dominant Hand *Mdn*: MT1: 91.9%, MT2: 94.7%, FT1: 98.1%, FT2: 98.1%; Play Dominant Foot *Mdn*: MT1: 74.5%, MT2: 94.5%, FT1: 94.7%, FT2: 88.2%). There were no consistent differences between tier groups or genders in relation to dominant side use, but top tier male players were generally less reliant on the dominant limb than were female players from both tiers. In general, top tier male players performed more successfully than either second tier male players or female players in both tiers. Most skills were executed under conditions of low opponent proximity, limiting the requirement for participants to use their non-dominant limbs. These findings illustrate the demands of Gaelic football in relation to bilateral skills, and we identified new research questions for future investigators.

## Introduction

Bilateral skill symmetry is the degree to which an individual can successfully perform with both sides of the body (Stöckel & Weigelt, 2012); it is widely believed to be a key characteristic of high-level sports performers (Moore, O’Dwyer, et al., 2017; Parrington et al., 2015). Indeed, Stöckel and Weigelt (2012) suggested that a certain degree of bilateral competence is essential if athletes are to be successful in modern team sports. This view has been endorsed by high performance Gaelic football players and coaches (Dillon et al., 2022), and Marcori et al. (2021, p. 382) noted that the advantage in being able to use both sides of the body in a sporting context "cannot be overstated” for team invasion sports, because this ability allows the athlete to adapt and remain accurate in their performance under varying task and environmental constraints.

Several investigators have explored the extent to which professional players utilize both limbs. Carey et al. (2001) found that professional players from the 1998 World Cup used their dominant foot in approximately 80% of their passing, dribbling, receiving and shooting actions. In a study of players from five national European leagues, Marcori et al. (2021) similarly found that left footed players performed 81% of their shots with their dominant foot, while right footed players performed 75% of their shots with their dominant foot. Stöckel and Weigelt (2012) extended previous research by examining how level of basketball play was related to bilaterality; they found that players made more use of their non-dominant hand as their caliber of play increased (11.5% in amateurs, 21.4% in semi-professionals, and 26.3% in professionals). These findings in soccer (Carey et al., 2001; Marcori et al., 2021) and basketball (Stöckel & Weigelt, 2012) led these respective authors to conclude that few players demonstrated bilateral play, even (or perhaps especially) at the professional level.

There are two methods of detecting a player’s laterality: direct observation via performance analysis within competitions (Carey et al., 2001, 2009; Giovanini et al., 2020; Stöckel & Weigelt, 2012), and player-completed questionnaires such as the Edinburgh Handedness Index (Oldfield, 1971) and the Waterloo Footedness Questionnaire (Elias et al., 1998). These two methods have produced varying results. For example, most professional soccer players surveyed by Grouios et al. (2002) self-reported mixed footedness (44.8%, with 36.9% right foot dominant and 18.3% left foot dominant), while Carey et al.’s (2009) notational analysis of professional soccer players reported 77.2% right foot use. Top level players from European basketball leagues self-reported mixed handedness (12.9%) (Stöckel & Vater, 2014), but Stöckel and Weigelt’s (2012) notational analysis of top European basketball players found that only 4.4% of the players demonstrated mixed handedness in game play. Thus, respondents’ self-assessments have tended to overstate their degree of mixed handedness/footedness when compared to notational analysis through direct observations of game play.

Another factor to consider is how situational demands, such as the proximity of an opponent player or a soccer player’s position on the pitch, may influence a player’s response with respect to bilaterality (Marcori et al., 2021; Stöckel & Vater, 2014). The limb a player uses in any given situation appears to result from the player’s interactions, skill and environmental context (Balagué Serre et al., 2019; Stöckel & Vater, 2014). In a study of high-performance soccer players in Europe’s top five leagues, Marcori et al. (2021) found that use of the dominant foot for shooting increased with an athlete’s distance from the target. The same authors found that shots taken from the right side of the pitch were performed more often with the left foot and vice-versa. Giovanini et al. (2020) concluded that game-to-game psychological pressures (e.g., play-off games vs. regular season games) affected NBA players’ limb choices. The general influence of an opponent’s proximity on task performance is well established. For example, McGuigan et al. (2021) reported that, when distance and angle to the target were held constant, close proximity of an opponent reduced the probability of a score in high performance Gaelic football. Additionally, in basketball, close proximity of opposition defenders negatively impacted shot success in several basketball studies (Alvarez et al., 2009; Ibáñez et al., 2008; Gorman & Maloney, 2016). While physical demands (e.g., further shooting distance; Marcori et al., 2021) and psychological demands (e.g., game importance; Giovanini et al., 2020) have been shown to influence limb use, most notational analyses of player laterality have not considered the effect of opponent proximity (e.g., Carey et al., 2001, 2009; Stöckel & Weigelt, 2012).

An important caveat in previous research on bilateral skill has been an exclusive focus on male players. In a review of publications on all topics from six sport and exercise journals across a 6-year period, Cowley et al. (2021) reported that female players comprised only 34% of 12,511,386 research participants. More studies of women are needed in sports generally and in athletes’ laterality specifically, because mixed handedness has been slightly higher in the general population for males (10.84%) than females (8.53%) (Papadatou-Pastou et al., 2020). Additionally, with respect to training activities during youth development, Ford et al. (2020) found that the mean number of accumulated hours in all soccer activity was lower among female international soccer players than among German male national team players. As bilateral skill can be developed through training (Haaland & Hoff, 2003), gender differences in training history may influence level of bilaterality seen among adults in high performance sport. Clearly, there is a need to compare female and male Gaelic players to ascertain whether gender-based differences in bilaterality exist in this group.

Gaelic football is an ideal natural laboratory for the investigation of bilateral skill symmetry, this sport permits use of both hands and feet (from both sides of the body) for completing the multiple skills involved in gaining, carrying and distributing the ball in attempts to score. Furthermore, given the importance to success of the efficient use of ball possession in Gaelic football (McGuckin et al., 2021), being bilaterally skilled would be logically advantageous. Our purpose in the present study was to investigate, via notational analysis of competitive matches, how performance level and gender influenced bilaterality among high performance male and female Gaelic footballers. Specifically, our first aim was to establish the extent to which the non-dominant side was used, and our second aim was to establish players’ success rates with both the dominant and non-dominant sides. As the opponent’s proximity is an important constraint on the decision to use a particular side of the body, our third aim was to establish the frequency of skill execution when players are within close proximity of an opponent.

## Method

### Participants

We acquired player video footage through freely available televised recordings and YouTube streams of games played in the National League prior to the Championship (All-Ireland) between 2017 and 2021. As this footage was in the public domain, our institutional ethics committee waived a requirement for participant consent when they reviewed and approved our study. All results are reported anonymously. Teams were ranked by tiers, based on their National League positions when games took place. The National League is a competition which takes place in spring of each year before the main Championship (All-Ireland) competition. In the male and female games, teams are divided into four league tables of eight teams each, with Division One being the highest and Division Four being the lowest. Teams in Division One and Two were categorised as Tier One county, whilst teams in Division Three and Four were categorised as Tier Two.

In total, we coded 121 games for males and females(76 male, 45 female). This equated to 88 hours of male games and 45 hours of female games (a female game lasts 60 minutes and a male game lasts 70 minutes). At least one action was coded for 696 players (181 from male Tier 1 (MT1) players, 238 from male Tier 2 (MT2) players, 181 from female Tier 1 (FT1) players and 115 from female Tier 2 (FT2)players). As detailed in the next section, we only included participants in our final analysis who had completed sufficient skill executions for a representative skill profile to be demonstrated. Table 2 illustrates that the sample size ranged from 114 players (when analyzing scores from play) to 310 players (when analyzing them solo). These players came from 23 different Irish county teams at Tier 1 (*n* = 7 male, 4 female) and Tier 2 (*n* = 6 male, 6 female).

### Research Design and Procedures

We chose a cross-sectional research design for comparing players’ bilateral abilities (as objectively identified) and their success rates when executing skills. The first author (with extensive coding experience) coded video footage using Nacsport performance analysis software (Nacsport Scout Plus). Table 1 provides an overview of what was coded, and the definition of each factor.

**Table 1.** Skill Definitions.

Skill	Successful definition	Unsuccessful definition
Hop	The player successfully bounces the ball and retains possession	The player attempts to bounce the ball but does not recover the possession
Solo	The player successfully solos the ball and retains possession	The player attempts to solo the ball but does not regain possession
Hand pass	The hand pass is gathered by the intended recipient of the same team. The ball starts in the players’ hands and is passed by striking the ball with one hand while holding the ball in the other	The intended recipient of the pass does not receive it due to an interception, a ball to the ground, or too high/low
Kick pass	The kick pass is gathered by the intended recipient	The kick pass missed its intended recipient
Pick up	Player successfully picks the ball off the floor and takes control of the ball in their hands	Player attempts to pick the ball off the floor but does not secure the ball in their hands
Shot for goal	A player in control of the ball takes a shot and it goes under the cross bar and into the goal	Ball is off target, goes wide, is saved, or blocked, by an opponent
Shot for point	Player in control of the ball attempts a shot for a point which is successful (goes over the bar)	Ball goes wide/short or is blocked down
Free kick or 45	Player attempts a shot for a point which is successful (goes over the bar) from a free kick	Ball is wide or short or stopped by an opposition player
		
Proximity of opponent – From the point the skill begins		
Close proximity	There is physical contact with the player at any point during the skill being executed, or the defender is close enough that they could affect the skill outcome (within arm’s reach)	
Out of reach	No opposition player within arm’s reach; the player with the ball does not need to adjust their actions as a result of an opponent	
	
Right/Left		
Skill	The side the skill is performed on. Hand the ball is dropped from	
	Hop – Hand the ball is dropped from	
	Solo – Foot the ball taps the ball with	
	Pick up – Foot the ball is picked up with	
	Hand pass – Hand the ball strikes the ball with	
	Shot – Foot the ball strikes the ball with	

A player in possession of the ball in Gaelic football can (a) distribute the ball, using either a hand pass or a kick pass, (b)carry the ball forward, using either a hop (bounce) or a solo dribble (kick the ball to oneself), or (c) attempt to score by kicking or hand passing the ball over the crossbar of the goals (a point) or by kicking the ball into the net (a goal). We developed a definition of a successful performance of each skill through conversations amongst our research team (see Table 1). In prior research, the definition of close proximity of an opponent was somewhat arbitrary; for example, both Corbett et al. (2018) and McGuigan et al. (2021) used “within 3m of a player on the ball.” Our definition, instead, was based on Gentile’s (1972, 2000) concept of regulatory features; if an opponent was sufficiently close for a player to have to alter (regulate) their movement to avoid being tackled, then that opponent was deemed to be within close proximity. In Gaelic Football, if an opponent was within arm’s reach of a player’s left-hand side, they had the potential to intercept the ball if it was bounced on that side. However, if the player bounced the ball on the right-hand side (i.e., the side opposite the opponent), then the action was performed too far away for the opponent to interfere. For all skills, if an opponent was sufficiently close to a player’s right-hand side that the player would be more likely to maintain possession if they performed the skill with the opposite (left) limb, the opponent was deemed to be in close proximity (See Table 1 and Supplemental File 1 for definition and illustration). This inference is consistent with the finding that when basketball skills are performed with opponents in close proximity, players typically use the most appropriate (and not necessarily the dominant) hand in that situation (Russo et al., 2019; Stöckel & Vater, 2014). The dominant limb was denoted via the limb used in the majority of a player’s skill executions.

We created a coding template in Nacsport that we used to register each player’s actions in a match, including the skill of interest and its associated descriptors as outlined in Table 1. The analyst watched each game, with the footage paused, slowed, or replayed to maximize the integrity of data collection. Once these data were exported for each match, they were transferred into a Master MS Excel 365 workbook containing all actions for the players. Each player was given a universal ID, and each game was given a code to denote the teams playing and the date of the match. Both the player ID and game codes enabled all of the data to be collated in an Excel file to allow for comparisons across multiple games for the same player. There were 48,336 actions coded between all 121 games.

Although a player may have used either limb on any particular skill execution, over the course of a series of games, their performance tended to demonstrate a stable profile (Hughes et al., 2001). We used Hughes et al.’s (2001) the limits of error method to establish the number of trials necessary to provide a representative profile for each skill. By representative profile, we mean a number large enough that including further skill executions would not change a player’s cumulative score by greater than ±5% of the final value. We applied this method to rating a subset of 30 players who had completed a minimum of 50 skill executions for the hop, solo, and handpass, and a minimum of 20 skill executions for the kick pass. In order to optimize the sample size and the average number of trials required for a player’s performance to stabilize assessment within ±5% of the final value chosen. Consequently, only players who had had executed 10 kick passes, 13 solos, 22 hops and 26 handpasses were included. The number of participants who achieved the minimum number of trials for analysis within each skill is shown in Table 2 in the results section. For each skill, we identified the percentage of skill executions completed with the right and left side. Thus, each player contributed one score to the analysis of dominant side use (their overall percentage dominance), irrespective of how many trials were used to generate that percentage.

**Table 2.** Player Percentages of Dominant Side Skill Use.

		Kick pass	Solo	Hand pass	Hop	Scores Play
		*p* < .001	*p* < .001	*p* < .001	*p* < .001	*p* < .001
Group		H(3) = 19.196	H(3) = 67.992	H(3) = 49.812	H(3) = 22.850	H(3) = 22.249
MT1	*Mdn*	90^a,b,c^	95.7^b,c^	83.7^a,b,c^	91.3b,c	74.5^a,b,c^
	Min	51.5	50	52.4	51.5	50
	Max	100	100	100	100	100
	n	71	95	88	77	35
	IQR	18.6	14.8	21.28	21.4	26
MT2	*Mdn*	92.8	96.6^d,e^	90.6^d,e^	94.5	94.5
	Min	53.8	50	50	52.2	70
	Max	100	100	100	100	100
	n	63	91	80	76	28
	IQR	10.0	13.0	16.2	19.92	15.4
FT1	*Mdn*	100	100	95.5	98.1	94.7
	Min	50	68.4	56.5	69.2	53.3
	Max	100	100	100	100	100
	n	44	62	63	41	27
	IQR	9.5	0.0	7.4	8.3	14.7
FT2	*Mdn*	100	100	95.7	98.1	88.2
	Min	72.7	52.6	51.7	62	51.5
	Max	100	100	100	100	100
	n	36	62	62	55	24
	IQR	7.8	0.0	7.9	6.9	15.2

*Note.* MT1 = Male tier 1; MT2 = Male tier 2; FT1 = Female tier 1; FT2 = Female tier 2; *Mdn* = Median; Min = Minimum; Max = Maximum; N = Total number of players within this cohort; n = number of players who had completed the minimum number of skill executions for inclusion; IQR = Interquartile Range; a = Significant difference between MT1 and MT2; b = Significant difference between MT1 and FT1; c = Significant difference between MT1 and FT2; d = Significant difference between MT2 and FT1; e = Significant difference between MT2 and FT2.

### Data Reliability

We insured data fidelity through training and reliability checks (McKenzie & van der Mars, 2015). To assess coding reliability we computed inter-rater reliability between the coding by the first author and another coder trained with the same coding system. The same set of definitions and coding window were used to tag a randomly selected full match. We calculated inter-rater agreement for each category by summing the number of agreed upon tags for each category and dividing by the total number of tags from each coder. We displayed that agreement as a percentage, with interrater agreement of 80% deemed acceptable (McKenzie & van der Mars, 2015; Van der Mars, 1989). Our inter-rater agreement ranged from 81.1%–86.7% for the categories of skill executed, limb choice, opposition proximity and success.

### Dominant versus Non-Dominant Side Success

No clear pattern of results was evident when comparing overall success with the dominant versus non-dominant limb. Within MT1, % success was higher with the dominant limb for the solo skill (z = 2.688; *r =* -.138; *p* = .007) and shots taken (z = −2.650; *r =* -.171; *p* = .008). Within MT2, % success was higher with the non-dominant hand for the hand pass (z = 7.832; *r =* .009; *p* < .001), and with the dominant leg for the kick pass (z = 5.905; *r =* .492; *p* < .001). Within FT1, % success was higher with the dominant hand for the hand pass (z = −6.359; *r =* -.253; *p* < .001), with the non-dominant leg for the kick pass (z = 5.580; *r =* .434; *p* < .001), and with the dominant hand for the hop (z = 1.990; *r =* .104; *p* = .047). Within FT2, % success was higher with the non-dominant leg for the kick pass (z = −3.201; *r =* .057; *p* = .001).

### Statistical Analysis

We conducted all statistical analyses using SPSS for Windows (IBM SPSS Statistics 28). We assessed data distributions for normality using visual analysis and Shapiro-Wilk tests. As we found these data skewed, we ran nonparametric inferential tests. The Kruskal–Wallis test identified any differences between the four tiers in the percentage of players’ dominant side use, percentage of skills preformed under close proximity of opponents and the percentage success on the dominant and non-dominant sides. We set statistical significance at *p* ≤ .05. Where significant differences were found, we conducted follow up Mann Whitney U tests for pairwise comparisons. To control family wise error (i.e., the probability of a false positive when making multiple comparisons), we employed the Holm-Bonferroni procedure (Holm, 1979) in which Bonferroni adjusted *p*-values were obtained by ordering *p*-values from lowest to highest. The smallest *p*-value was then multiplied by k, where k was the number of hypotheses to be tested. The resulting *p*-value was considered to be statistically significant if it was less than 0.05. The next smallest *p*-value was then multiplied by k-1, and again compared to 0.05, with this process continued until a null hypothesis was accepted. We calculated Wilcoxon Signed Ranking tests to ascertain whether there was a significant difference within tiers between dominant and non-dominant limb success when executing skills.

## Results

### Percentage of Dominant Side Skill Execution

Players’ percentages of skill use on the dominant side are illustrated in Table 2. Median values indicated that players in all groups predominantly used their dominant side to execute the kick pass. However, inspection of the minimum values in Table 2 illustrates that some players were bilateral (i.e., used dominant limb at a rate close to 50%).

Kruskal Wallis tests identified a significant difference between all four tier/gender groups for the kick pass, H(3) = 19.196, *p* < .001, and follow up Mann Whitney U post-hoc tests revealed three pairwise comparisons that showed a significant difference: MT1 (*Mdn* = 90%, IQR = 18.60%) and FT1 (*Mdn* = 100%, IQR = 9.5%; z = −3.471; *r =* -.212; *p* < .001) with FT1 higher, MT1 and FT2 (*Mdn* = 100%, IQR = 8.15%; z = −3.641; *r =* .077; *p* < .001) with FT2 higher.

Median values for the solo skill again indicated that most skill executions were with the dominant side across all four tier groups (Table 2), whilst a Kruskal–Wallis test identified a significant difference in dominant solo use between MT1, MT2, FT1 and FT2; H(3) = 67.992, *p* < .001. Follow up Mann Whitney U tests revealed four pairwise comparisons that showed a significant difference: MT1 and FT1(*z =* -6.261; *r =* -0.126; *p* < .001) with FT1 higher in dominant side use. MT1 and FT2 (*z =* -703; *r =* -0.127; *p* < .001) with FT2 higher, MT2 and FT1 (*z =* -5.919; *r =* -0.060; *p* < .001) with FT1 higher, MT2 and FT2 (*z =* -5.366; *r =* -0.158; *p* < .001) with FT2 higher.

Kruskal Wallis tests identified a significant difference in dominant hand use for the handpass across MT1, MT2, FT1 and FT2, H(3) = 49.812, *p <* .001. Mann Whitney U tests revealed five pairwise comparisons that showed a significant difference: MT1 and MT2 (*z =* -3.891; *r = -*.130; *p* < .001) with MT2 higher in dominant side use, MT1 and FT1 (*z =* -6.281; *r =* .199; *p* < .001) with FT1 higher, MT1 and FT2 (*z =* -5.456; *r =* -0.171; *p* < .001), with FT2 higher, MT2 and FT1 (*z =* -2.585; *r =* .199; *p* = .030), with FT1 higher.

Kruskal Wallis tests identified a significant difference in dominant hand use across MT1, MT2, FT1 and FT2 for the hop, H(3) = 22.850, *p <* .001. Follow up Mann Whitney U tests showed that significant differences occurred in two pairwise comparisons: MT1 and FT1 (z = −3.932; *r =* -0.345; *p <* .001), with FT1 higher, and MT1 and FT2 (*z =* -4.007; *r =* -0.360; *p* < .001), with FT2 higher.

Kruskal Wallis tests identified a significant difference in dominant leg use across MT1, MT2, FT1 and FT2 for shot-taking, H(3) = 22.249, *p <* .001. Follow up Mann Whitney U tests showed that significant differences only occurred between MT1 and MT2 (z = −4.347, *r =* -.883, *p* < .001), with MT2 higher, MT1 and FT1 (z = −3.550, *r =* .203, *p* < .001), with FT1 higher, and MT1 and FT2 (z = −2.942, *r =* -.169, *p =* .042), with FT2 higher.

### Success from Dominant or Non-Dominant Side Use

An overview of results on skill execution success is provided in Table 3. Kruskal Wallis tests identified a significant difference in kick pass success with dominant leg use across MT1, MT2, FT1 and FT2; H(3) = 26.143, *p <* .001. Follow up Mann Whitney U tests showed that significant differences occurred in three pairwise comparisons: MT1 and FT1 (*z =* 2.723, *r =* .111; *p* = .006) with success greater in MT1, MT1 and FT2 (*z =* -5.036; *r =* .038; *p* < .001) with success greater in MT1, and MT2 and FT2 (*z =* 2.504; *r =* .211; *p* = .012) with success greater in MT2.

**Table 3.** Success With Dominant and Non-dominate Side Use for Each Skill by Tier/Gender Group.

Skill		Kick pass		Solo		Hand pass		Hop		Scores Play	
		*p <* .001	*p* < .001	*p <* .001	*p =* .014	*p <* .001	*p* = .012	*p <* .029	*p =* .450	*p =* .164	*p =* .508
		H(3) = 26.143	H(3) = 22.585	H(3) = 18.010	H(3) = 10.622	H(3) = 13.504	H(3) = 10.907	H(3) = 9.053	H(3) = 2.644	H(3) = 5.115	H(3) = 2.322
		Dom	non-dom	Dom	non-dom	Dom	non-dom	Dom	non-dom	Dom	non-dom
MT1	*Mdn*	91.9^b,c^	100	100^c^	100^c^	97.3^c^	100	100^c^	100	62.0	33.3
	Min	66.7	50	89.7	94.40	88.20	0	92	50	21.4	0.0
	Max	100	100	100	100	100	100	100	100	85.7	100
	n	71	65	95	60	88	93	77	65	35	30
	IQR	9.0	14.3	2.0	0.0	4.8	6.75	2.4	0.0	18.45	40.7
MT2	*Mdn*	100^e^	100	100^d,e^	100^e^	97.6^d,e^	100	100	100	64.7	50
	Min	86.75	0.0	92.9	75	89.7	83.3	91.7	75	10	0.0
	Max	100	100	100	100	100	100	100	100	88.9	100
	n	63	29	91	63	80	53	76	43	28	16
	IQR	11.1	22.33	2.1	0.0	5.0	0	3.15	0.0	36.7	62.5
FT1	*Mdn*	82.40	100	100	100^f^	96	96	100	100	57.1	25
	Min	54.5	0.0	53	100	83.3	0	86	50	23.8	0.0
	Max	100	100	100	100	100	100	100	100	100	100
	n	44	13	62	11	63	59	41	25	27	17
	IQR	12.6	37.53	4.38	00	5.4	0	3.4	0.0	30.2	62.5
FT2	*Mdn*	78.25	79.5	97.5	100	95.5	100	98.3	100	54.9	50
	Min	46.2	0.0	88.2	75	83.3	0	85	0.0	23.1	0.0
	Max	100	100	100	100	100	100	100	100	76.5	100
	n	36	19	62	13	62	62	55	28	24	20
	IQR	21.80	31.2	5.3	2.9	4.3	7.65	3.8	0.38	30.5	57.5

*Note.* MT1 = Male tier 1; MT2 = Male tier 2; FT1 = Female tier 1; FT2 = Female tier 2; *Mdn* = Median; Min = Minimum; Max = Maximum; N = Total number of players within this cohort; n = number of players who had completed the minimum number of skill executions for inclusion; IQR = Interquartile Range; Dom = Dominant side; Non-Dom = Non-dominant side. H = Kruskal-Wallis; p = *p* value comparing between skills;

^a^ = Significant difference between MT1 and MT2

^b^ = Significant difference between MT1 and FT1

^c^ = Significant difference between MT1 and FT2

^d^ = Significant difference between MT2 and FT1

^e^ = Significant difference between MT2 and FT2

^f^ = Significant difference between FT1 and FT2.

With respect to non-dominant leg use, Kruskal Wallis tests identified a significant difference in kick pass success across the four groups; H(3) = 22.585, *p<*.001 Follow up Mann Whitney U tests showed that significant differences occurred in two pairwise comparisons: MT1 and FT2 (*z =* 4.493, *r* = .464; *p* < .001) with success greater in MT1, and MT2 and FT2 (*z =* 3.745; *r = -.270*; *p* < .001) with success greater in MT2.

Kruskal Wallis tests identified a significant difference in the success of dominant leg use for solo across MT1, MT2, FT1 and FT2, H(3) = 18.010, *p <* .001. Follow up Mann Whitney U tests showed that significant differences occurred in three pairwise comparisons: MT1 and FT2 (*z =* 2.571; *r =* -.233; *p* = .0) with MT1 higher, MT2 and FT2 (*z =* 4.036; *r =* .187; *p* < .001) with MT2 higher, and MT2 and FT1 (*z =* 2.641; *r =* -.460; *p* = .040), with MT2 higher. Despite the similarity in the group median scores, significant differences arose due low levels of variance within groups.

Kruskal Wallis tests also identified a significant difference in the success of non-dominant leg use for solo across MT1, MT2, FT1 and FT2, H(3) = 10.622, *p =* .014. Follow up Mann Whitney U tests showed that significant differences occurred in three comparisons: MT1 and FT2 (*z =* 3.074; *r = .379*; *p* = .012) with MT1 higher, MT2 and FT2 (*z =* 2.892; *r = -*0.284; *p* = .020) with MT2 higher, and FT1 and FT2 (*z =* 2.892; *r = -*0.381; *p* = .036), with FT1 higher.

Kruskal Wallis tests identified a significant difference, in the success of dominant hand pass across MT1, MT2, FT1 and FT2; H(3) = 13.504, *p <* .001. Follow up Mann Whitney U tests showed that significant differences occurred in two pairwise comparisons: between MT1 and FT1 (*z =* 3.081; *r =* .295 *p* = .012) with MT1 higher, MT2 and FT2 (z = 3.357; *r = -*.022; *p* < .001) with MT2 higher.

While the Kruskal–Wallis test identified a significant difference in the success of the non-dominant hand for a pass across the four groups (H(3) = 10.907, *p =* .012), following the Holm Bonferroni correction, none of the follow up Mann Whitney U tests showed significant differences between the paired groups.

Kruskal Wallis tests identified a significant difference H(3) = 9.053, *p =* .029 in the success of the dominant hand for hop across MT1, MT2, FT1 and FT2. Follow up Mann Whitney U tests showed that significant differences occurred in one comparison: between MT1 and FT2 (*z =* 2.7773; *r =* .067; *p* = .036) with MT1 higher. Kruskal Wallis tests identified no significant differences in the success of the non-dominant hand for hop across MT1, MT2, FT1 and FT2, H(3) = 2.644, *p =* .450.

Kruskal Wallis tests identified no significant difference in the success of shots taken in play across the four groups with either the dominant leg (H(3) = 5.115, *p =* .164) or the non-dominant leg (H(3) = 2.322, *p =* .508).

### Percentage of Skills Executed Under Close Opposition Proximity

Table 4 shows that most skills were not executed in close proximity to an opponent. For the kick pass (H(3) = 5.437, *p* = .142), solo (H(3) = 5.879, *p =* .118), and hop (H(3) = 6.513, *p* = .089)) there were no significant differences between the four groups in the percentages of skills executed in close proximity of an opponent.

**Table 4.** Percentage of Skills Executed Within Close Proximity of an Opponent.

Group		Kick pass	Solo	Hand pass	Hop	Scores Play
		*p =* .142	*p =* .118	*p* < .001	*p =* .090	*p =* .001
		H(3) = 5.437	H(3) = 5.879	H(3) = 32.559	H(3) = 6.491	H(3) = 15.691
MT1	*Mdn*	11.1	4.9	15^b,c^	13.30	60^c^
	Min	0.0	0.0	0.0	0.0	10
	Max	50	24.1	41.8	68.8	100
	n	71	95	88	77	35
	IQR	13.1	7.7	9.28	14.1	47
MT2	*Mdn*	9.1	4.25	13.1^d^	12.05	27.3
	Min	0.0	0.0	0.0	0.0	10.0
	Max	36.4	20	33.3	100	75
	n	63	91	80	76	28
	IQR	15.4	6.7	8.8	11.1	32.48
FT1	*Mdn*	11.1	3.9	9	13.6	28
	Min	0.0	0.0	1.8	0.0	0.0
	Max	31.8	22.2	30.2	40.4	80
	n	44	62	63	41	27
	IQR	13.85	7.7	7.3	11.4	37.8
FT2	*Mdn*	12.2	4.3	10.5	15.8	29.45
	Min	0.0	0.0	0.0	0.0	7.1
	Max	55.6	31.8	40.7	100	80
	n	36	62	62	55	24
	IQR	18.45	9.1	9.4	16.1	19.08

*Note.* MT1 = Male tier 1; MT2 = Male tier 2; FT1 = Female tier 1; FT2 = Female tier 2; *Mdn* = Median; Min = Minimum; Max = Maximum; N = Total number of players within this cohort; n = number of players who had completed the minimum number of skill executions for inclusion; IQR = Interquartile Range

^a^ = Significant difference between MT1 and MT2

^b^ = Significant difference between MT1 and FT1

^c^ = Significant difference between MT1 and FT2

^d^ = Significant difference between MT2 and FT1

^e^ = Significant difference between MT2 and FT2

^f^ = Significant difference between FT1 and FT2.

Kruskal Wallis tests identified a significant difference H(3) = 32.559, *p <* .001 in the proximity of opponents for the hand pass across MT1, MT2, FT1 and FT2. Follow up Mann Whitney U tests showed significant differences between three pairwise comparisons: MT1 and FT1 (z = 5.248; *r =* .048; *p <* .001) for which MT1 had more close opponents, MT1 and FT2 (z = 3.564, *r* = −.050, *p* < .001) for which MT1 had more close opponents, and MT2 and FT1 (z = 3.879; *r =* .036; *p* < .001) for which MT2 had more close opponents.

Kruskal Wallis tests identified a significant difference H(3) = 15.691, *p =* .001 in the proximity of opponents in relation to shots taken across the groups MT1, MT2, FT1 and FT2. A follow up Mann Whitney U test showed that significant differences occurred in three pairwise comparisons: MT1 and MT2 (z = 8.375; *r* = .085; *p* < .001) with MT1 showing more close opponents, MT1 and FT1 (z = 2.806*;r* = .525,*p* = .005) with MT1 showing more close opponents, MT1 and FT2 (z = 3.118; *r = -.*448; *p* = .002), with MT1 showing more close opponents

## Discussion

Our aim of this study was to investigate how frequently male and female Gaelic footballers of varying skill levels used their non-dominant side on sport skill tasks, how much success they had with these skills when using either side, and the frequency with which opponents were in close proximity when they executed these skills (kick pass, hand pass, hop, solo, and shots). Irrespective of players’ playing tiers or gender, intercounty players primarily used their dominant side when executing skills. There were minimal differences between tiers in terms of non-dominant side use. In terms of success percentages, findings were more mixed. For example, five skill/tier combinations showed higher success on the dominant side, and three combinations showed higher success with the non-dominant side, with remaining comparisons showing no difference. Most skill executions were not in close proximity to an opponent, suggesting limited necessity for using one side or another; except that, for shooting, an exception was shooting, where MT1 players were more commonly executing skills in close proximity to an opponent.

The finding that high performance Gaelic football players predominantly relied on their dominant side for most skill executions is consistent with previous research on male athletes in other sports (Carey et al., 2001, 2009; Moore et al., 2017; Stöckel & Vater, 2014; Stöckel & Weigelt, 2012). Differences between skills were generally small, consistent with Carey et al.’s (2001) analysis of various soccer skills executed during open play (i.e., not set piece skills). This consistency in bilaterality contrasts with basketball findings for certain skills, where professional players were observed to use the dominant side 54% of the time for dribbling while still relying primarily on the dominant side for shooting (79% dominant side) and passing (81% one handed passes used dominant side) (Giovanini et al., 2020). Stöckel and Vater (2014) hypothesised that extensive practice may develop bilaterality in skills which are routinely performed in close proximity to opponents, such as when dribbling and shooting layups in basketball. Despite the intuitive benefit of bilaterality for Gaelic football, variations in game conditions such as weather and pitch conditions and the larger size of the playing area may inhibit (or at least not promote) the development of bilaterality in Gaelic footballers. Future researchers should investigate whether variations in game conditions, or differences in the amount and quality of bilateral practice, are responsible for the lower incidence of bilaterality among Gaelic footballers.

Within tiers, we observed mixed differences in dominant limb use, and there were no patterns that fit a priori hypotheses such as MT1 showing less dominant limb use across all skills than MT2, and FT1 showing the same versus FT2. For example, MT1 and MT2 differed on three comparisons (two in favor of MT1, one in favor of MT2), while two comparisons revealed no difference and FT1 and FT2 did not differ in any comparisons. Nevertheless, there was a clear difference between the levels of bilaterality seen in MT1 compared to FT1 and FT2 (MT1 greater in 9/10 comparisons). MT2 was greater than FT1 and FT2 in only 2/10 comparisons, and MT2 used the dominant side more in one comparison (the handpass). While top tier males used their non-dominant limbs more than females, there were fewer differences between second tier males and either tier of females. What remains unclear is whether MT1 participants’ reduced reliance on the dominant side for two skills, and MT2 participants’ reduced reliance on the dominant side for one skill resulted from practice or “an advanced selection process” (i.e., the tendency for coaches to have to more often selected naturally mixed-handed players) as has been hypothesised in research with professional basketball players compared to semi-professional and amateur players (Stöckel & Vater, 2014). There is a need for investigators to delve further into players’ developmental histories in each tier, to investigate and compare players with varied practice history, competition history, and participation in other sporting activities (Hopwood, 2013).

As this study is the first performance analysis study to include female athletes in a measurement of bilaterality, we were unable to compare levels of bilaterality we found to females in other sports. We cannot yet say why our top tier female players were largely less bilateral in skill use than their top tier male counterparts. Perhaps this is due to a smaller practice volume accumulated by female players (Hornig et al., 2016), but there is currently no data to support this suggestion in Gaelic Football. There are, however, indications that female players engage in less soccer play (outside of formalized practice) during childhood (Hendry et al., 2019; Hodges et al., 2020), with one research team having estimated that academy-based, elite male youth soccer players in Scotland had already accumulated six times more play hours by age 16 years than the adult female National team players Hendry & Hodges (2018). Further study of practice and playing history relationships to bilaterality in male and female players is needed.

Within the eight comparisons which did show a difference between dominant and non-dominant success, three involved the kick pass. One reason for the kick pass differences is that effective kicking is associated with the ball being dropped on the foot with the hand on the same side as the kicking leg (Ball, 2008). The non-dominant hand is typically less consistent on the ball drop than the dominant hand (Ball, 2008). Consequently, players often avoid using two non-dominant limbs together (for kicking) by using the contralateral (opposite) hand or both hands together when dropping the ball to the non-dominant foot (Pavely et al., 2010). Coaches should be cognisant of the challenges inherent in using two non-dominant limbs in concert (i.e., dropping a ball from non-dominant hand to kick with non-dominant foot) when training younger players in particular. Researchers and Gaelic football coaches should explore different strategies to facilitate the learning of non-dominant side kicking (e.g., would players benefit from developing the non-dominant foot to kick from the ground (soccer style) before progressing to using the non-dominant hand to feed the ball to the non-dominant leg in a punt kick?).

Given a well-established bias towards using the dominant hand or foot (Carey et al., 2001, 2009; Moore et al., 2017; Stöckel & Vater, 2014; Stöckel & Weigelt. 2012), we would expect players to act against that bias when circumstances dictate (i.e., when an opponent is preventing the preferred use of the dominant side, when proximity to the pitch boundaries encourage use of a particular side, or when chances of success are increased by using the non-dominant side; Stöckel & Vater, 2014). If circumstances that induce a player to use their non-dominant side are rare, then the frequency of non-dominant side use should be rare. Given the rarity we uncovered with respect to close proximity of opponents in Gaelic football when executing three of the skills we analyzed, there is reason to question the importance of having the ability to use the non-dominant side for these three skills. Moreover, it is hard to use skill success as the determinant that a given choice was optimal, since non-dominant limb use, even if less successful on average, may have offered better tactical opportunities in a given game context (Dillon et al., 2022). Thus, future research into bilateral skill should measure both the appropriateness of decision making and the effectiveness of skill execution (Oslin et al., 1998).

The ability to perform skills bilaterality is widely believed to be a key characteristic of high-level sports performers because it increases the likelihood of players finding a solution for each scenario they face (Dillon et al., 2022; Moore, O’Dwyer, et al., 2017; Parrington & Ball, 2015; Stöckel & Vater, 2014). There is some support for this position in that Murray et al. (2023) found that bilateral Gaelic football players were more likely to be selected for a county under 17 team than their more unilateral counterparts. However, our finding that top level adult players rarely used the non-dominant limb contrasts with this view. To understand this apparent contradiction, Researchers should examine how bilaterality influences player decision making rather than simply skill execution. In addition, in future analyses researchers should focus on just those skill executions in close proximity to an opponent (i.e., where there is a distinct advantage to performing with one side or another) rather than all skill executions. Such research would offer valuable guidance to coaches in relation to the amount of practice that should be devoted to the development of bilateral skill.

### Limitations and Further Considerations for Future Investigators

Among this study’s limitations was an analysis based on a small number of skill executions under close proximity of an opponent. Future researchers should collect a greater number of trials from each player, focusing on situations where there is a distinct advantage to using one limb or the other due to opponent proximity or location on the pitch. Within this research, consideration should also be given to other factors that may influence a player’s choice of how to execute a skill such as the quality of the opposing player relative to the player executing the skill, the physical or mental condition of the player in that given moment, the score in the match, and the importance of the match.

A second limitation resulted from our focus on the players’ technical executions but not their associated decision-making. Players are often able to manipulate a situation to avoid using their non-dominant side. The extent to which lateral preference influenced player decision-making can be informative (Oslin et al., 1998) and would be an appropriate additional focus of future researchers.

A third limitation was our reliance on match footage as source data. While coding video footage had the advantage of capturing aspects of the competitive context, it is labor intensive and relatively few trials were recorded per player per match. Future researchers might also utilize small-sided game assessments (Bennett et al., 2018; Klingner et al., 2021) to get a more detailed understanding of how players utilize both sides of the body to adapt to opponents’ positioning. Such assessments may have an additional benefit for squads looking to evaluate their players’ levels of bilateral skills, given the high concentration and variety of skill executions quickly demanded in such games.

## Conclusion

This study is the first to record bilaterality in game play of male and female high-performance Gaelic footballers. We revealed that, irrespective of playing tier or gender, intercounty players primarily used their dominant side when executing skills. There were no consistent differences between groups formed on the basis of player skill tiers and genders, but the highest performance male players were generally less reliant on their dominant limb than either lower performance male players or female players at both tiers. Most skill executions were not in close proximity to an opponent, suggesting a limited need to use one side of the body or the other, except that shots taken by high performance male players were more often in close proximity to opponents. The frequency with which players executed skills without an opponent close enough to interfere should prompt reflection from coaches on the need to train players to use both sides of their body on all sport skills. To aid that reflection, future researchers should explore how bilaterality influences decision making by players with the ball.

## Supplemental Material

Supplemental Material - Profiling Bilateral Skills in High-Performance Male and Female Gaelic Footballers Supplemental Material for Profiling Bilateral Skills in High-Performance Male and Female Gaelic Footballers by K. Dillon, I. Sherwin, and P. E. Kearney in Perceptual and Motor Skills

## Data Availability

Data sharing not applicable to this article as no datasets were generated or analyzed during the current study.
