# Supplementary material for: Profiling Bilateral Skills in High-Performance Male and Female Gaelic Footballers
Source: Percept Mot Skills. 2024 Mar 23;131(3):920–39. doi: 10.1177/00315125241238307 (PMC11127500; doi:10.1177/00315125241238307)

Supplementary Material A

Out of Reach	Close Proximity
**No opposition player within arm's reach during the execution of the skill; the player with the ball does not need to adjust their actions because of an opponent.**<br><br>**In image (a), the player in possession can bounce the ball with either hand without an opponent being able to interfere. In this instance, it is appropriate to use either hand to bounce the ball.**	There is physical contact with the player at any point during the skill being executed, or the defender is close enough that they could affect the skill outcome (within arm's reach) depending on how the skill is executed.<br><br>In image (b), the opposition player has made contact with the player in possession and therefore is in Close Proximity.<br><br>In image (c), if the player in possession bounced the ball with their left hand, the opponent is close enough to reach out and potentially intercept. However, if the player in possession bounced the ball with their right hand, the player's body shield's the ball from their opponent. In this instance, it is more appropriate to use the right hand to bounce the ball. Regardless of what the player in possession does, this situation is coded as Close Proximity.

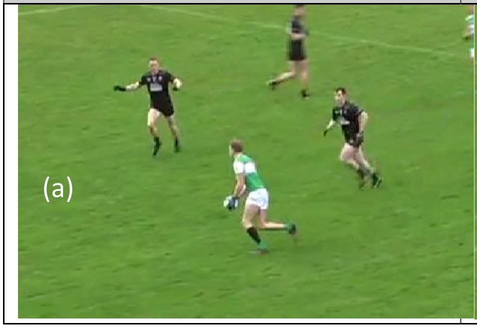

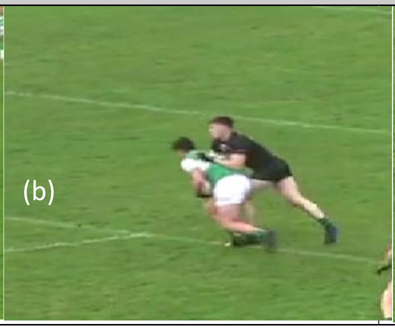

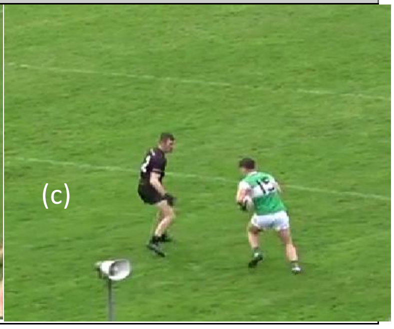

Supplement: Supplemental Material - Profiling Bilateral Skills in High-Performance Male and Female Gaelic Footballers [file sj-pdf-1-pms-10.1177_00315125241238307.pdf]
